# Emerging Molecular Technologies in Renal Cell Carcinoma: Liquid Biopsy

**DOI:** 10.3390/cancers11020196

**Published:** 2019-02-07

**Authors:** Alessia Cimadamore, Silvia Gasparrini, Francesco Massari, Matteo Santoni, Liang Cheng, Antonio Lopez-Beltran, Marina Scarpelli, Rodolfo Montironi

**Affiliations:** 1 Section of Pathological Anatomy, Polytechnic University of the Marche Region, School of Medicine, United Hospitals, Via Conca 71, I‐60126 Ancona, Italy; alessiacimadamore@gmail.com (A.C.); silvia.gasparrini@hotmail.com (S.G.); m.scarpelli@univpm.it (M.S.); 2 Division of Oncology, S.Orsola-Malpighi Hospital, 40138 Bologna, Italy; fmassari79@gmail.com; 3 Oncology Unit, Macerata Hospital, 62100 Macerata, Italy; mattymo@alice.it; 4 Department of Pathology and Laboratory Medicine, School of Medicine, Indiana University, Indianapolis, IN 46202, USA; liang_cheng@yahoo.com; 5 Department of Surgery, Medical School, Cordoba University, 14071 Cordoba, Spain; em1lobea@gmail.com

**Keywords:** renal cell carcinoma, circulating DNA, CTC, diagnosis, follow-up, genetic alteration, target therapy

## Abstract

Liquid biopsy, based on the circulating tumor cells (CTCs) and cell-free nucleic acids has potential applications at multiple points throughout the natural course of cancer, from diagnosis to follow-up. The advantages of doing ctDNA assessment vs. tissue-based genomic profile are the minimal procedural risk, the possibility to serial testing in order to monitor disease-relapse and response to therapy over time and to reduce hospitalization costs during the entire process. However, some critical issues related to ctDNA assays should be taken into consideration. The sensitivity of ctDNA assays depends on the assessment technique and genetic platforms used, on tumor-organ, stage, tumor heterogeneity, tumor clonality. The specificity is usually very high, whereas the concordance with tumor-based biopsy is generally low. In patients with renal cell carcinoma (RCC), qualitative analyses of ctDNA have been performed with interesting results regarding selective pressure from therapy, therapeutic resistance, exceptional treatment response to everolimus and mutations associated with aggressive behavior. Quantitative analyses showed variations of ccfDNA levels at different tumor stage. Compared to CTC assay, ctDNA is more stable than cells and easier to isolate. Splice variants, information at single-cell level and functional assays along with proteomics, transcriptomics and metabolomics studies can be performed only in CTCs.

## 1. Introduction

Liquid biopsy, based on the circulating tumor cells (CTCs) and cell-free nucleic acids (i.e., circulating cell-free DNA (ccfDNA), circulating tumor DNA (ctDNA), circulating cell-free RNA (ccfRNA) or extracellular vesicles (EVs) and their cargo) from blood and urine, has received great attention because of its potential tool for monitoring the disease status in patients with urogenital cancers, including renal cell carcinoma (RCC) [1]. Examining all these circulating biomarkers are beyond the scope of this review, which will focus on ctDNA in RCC.

The advantages of doing ctDNA assessment versus tissue based genomic profile are the minimal invasive procedures and the consequent minimal procedural risk, the possibility to serial testing in order to monitor disease-relapse and response to therapy over time and to reduce hospitalization costs during the entire process, from diagnosis to follow-up. From a pathological point of view, ctDNA is more likely to represent whole tumor instead that only one tumor section, it also permit to evaluate the possible changes in tumor genes expression and enlighten mechanisms underlying resistance to therapy [1]. Renal cell carcinoma is the sixth most frequently diagnosed cancer in men and the 10th in women. Clear cell RCC (ccRCC) is the most common subtype and *VHL* tumor suppressor gene is the most frequently mutated gene [2]. Comprehensive molecular characterization of RCC identified several mutations, including *PBRM1*, *SETD2*, *BAP1*, *KDM5C*, *TSC1*, *TSC 2* and *MTOR* [3]. The first three genes are located at 3p21 and function as tumor suppressors. Their mutations correspond to a loss of heterozygosity and a loss of their functions while *MTOR* mutations are generally functional activating. This could be the reason why patients with *MTOR* mutations respond to mTOR pathway inhibitors, such as everolimus and temsirolimus [4]. *PBRM1*, *SETD2* and *BAP1* as well as *KDM5C* mutations are associated with adverse outcomes [5]. Moreover, patients treated with sunitinib with *KDM5C* mutations have lower risk of progression compared to non-mutated patients [6]. Detection of these prognostic and predictive genetic alterations in a non-invasive way will improve clinical outcomes and quality of life of RCC patients. In this article, we discuss the recent findings and issues related to liquid biopsy in RCC, with a focus on circulating DNA, conducting a literature search of the following keywords: cell-free DNA; circulating-tumor DNA, liquid biopsy, renal cell carcinoma.

## 2. Critical Issues Related to ctDNA Assays

Genomic profiles of liquid biopsy have been shown to match very closely those of the corresponding tumors [7]; however, all ctDNA assays have a considerable rate of discordance with tissue testing. Except for cancer histotypes harboring a specific genetic alteration, ctDNA do not correlate directly with a histologic or cellular phenotype. Indeed, in lung cancer studies, using tissue genotyping as the reference standard, the sensitivities in finding a specific mutation is moderate (averaged 66%), while the specificity is usually very high (averaged 96%) [8,9,10,11,12]. Same results have been obtained by Hao and colleagues in a meta-analysis on 1812 patients with colorectal cancer [13]. The sensitivity of ctDNA assays in detecting ctDNA in the plasma depends on the assessment technique and genetic platforms used, on tumor-organ, stage, tumor heterogeneity, tumor clonality [14].

Commercial available platforms for assessing ctDNA detects distinct classes of genetic alterations (single nucleotide variants, insertions/deletions (indels), copy number amplifications, fusions) in different numbers of cancer-related genes. Some clinical research studies sequence specific genes of interest applying hotspots panels [15,16,17]. The assay can be targeted for a single or a small number of variants, or aiming for a broader coverage [18,19].

**Figure 1:**
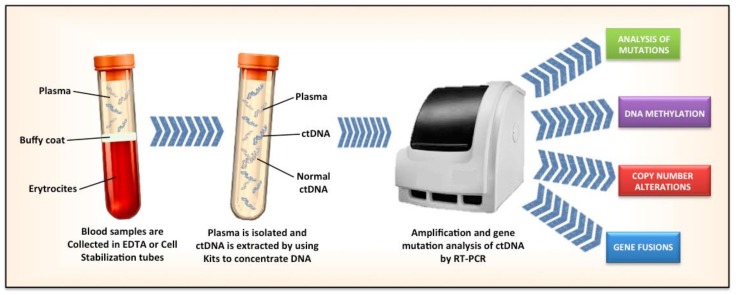
Steps in the isolation of cell tumor DNA in the blood.

The process of genomic testing start with a blood draw in two EDTA or Cell Stabilization tubes, DNA fragments undergo real-time or digital PCR or to Next Generation Sequence (NGS) analysis. The results are digital sequences analyzed to identify genetic alterations, quantitative mutant allele fractions and gene copy numbers. Different assays may have different lower limits of detection or interrogate different genomic regions (Figure 1).

Independently of analytical factors, several biological elements may affect the sensitivity of the test. It is demonstrated that the concentration of ctDNA increased with stage; in fact the fractions of patients with cancer of any type, with detectable ctDNA was 47%, 55%, 69% and 82% for patients with stage I, II, III and IV respectively [20]. It is also demonstrated that ctDNA levels decrease when a tumor is responding to therapy [21,22] and can be used as biomarker of response to therapy [23].

Regarding tumor site, detectable levels of ctDNA was found in more than 75% of patients with metastatic cancer of pancreas, bladder, colon, breast, liver, stomach, and in less than 50% of patients with metastatic RCC, prostate and thyroid tumor [20]. The absence of a specific therapy-driving gene alteration could preclude the possibility of a patient to be treated with a target therapy. The undetected results should be confirmed with analysis of tumor tissue sample. A diagnostic approach relying only on ctDNA analysis could fail to identify relevant information in driving patient treatment decisions. Presence of detectable ctDNA has been proposed as a fourth parameter in a modified staging system: TNMB. Paralleling the ‘M’ category, initial categorization may be defined as the absence (‘B0’) or presence (‘B1’) of detectable ctDNA [24].

## 3. Concordance between Tissue Based-Biopsy and ctDNA

Comparing genetic alterations (GA) detected by NGS in tumor tissue and ctDNA, Hahn et al. found that the median mutation rate for tissue NGS was 10.0 and for ctDNA was 2.2 but the concordance rate was only 8.6% [25]. Such results can be partially explained by presence of subclones, the time between tumor tissue and ctDNA NGS (mean time: 22 months, range 0–70 months) and by the fact that some patients (3 out of 19) received treatment in the period between the tests. Similar results were also obtained by Chae in 28 patients with advanced solid tumors, in which more than 50% of mutations detected in either technique were not detected using the other biopsy technique, proposing “a potential complementary role of each assay [26].”

In the study of Hahn, all of 19 metastatic RCC patients have GA detected on tissue based NGS while only 13 (68%) have GA at ctDNA NGS analysis. Six patients have no GA detected on ctDNA NGS assay. These so-called “negative” patients may comprehend patients with undetectable ctDNA in the metastatic phase, patients with GA not covered by the genetic platforms, or samples undergoing assay errors. Furthermore, among patients with GA detected by both biopsy methods, only five have the same mutations they had in the tissue-based NGS, all affecting *TP53* and *VHL* genes. *VHL* is a truncal mutation in metastatic RCC (mRCC) and its aberrations are conserved events across all clones. However, the concordance rate for *VHL* is only 50%. According to these results, it is quite unlikely to monitor disease progression or relapse with only one gene-specific test in the follow-up.

## 4. Qualitative Analyses of ctDNA

Pal and colleagues analyzed ctDNA in 220 patients with mRCC. 79% of patients had GA detected, the most frequent involving *TP53* (35%), *VHL* (23%), *EGFR* (17%), *NF1* (16%), and *ARID1A* (12%), with a preponderance of single nucleotide variants and small indels. They also compared GA detected in ctDNA analysis in first-line patients (sunitinib and pazopanib) and in later-lines patients (nivolumab, everolimus, axitinib, and cabozantinib). They note a higher frequency of *TP53* (49% vs. 24%, *p* = 0.02), *NF1* (20% vs. 3%, *p* = 0.01) alterations in later-line therapy group with subsequent VEGF-directed therapy. These findings raise the hypothesis that certain GAs may arise as a consequence of selective pressure from therapy and may have a role in therapeutic resistance [27].

Searching for specific mutations in molecular pathways known to be related to sensitivity to therapy is functionally relevant to guide treatment decision. Sensitivity to everolimus seems to be related to presence of *TSC1/TSC2/MTOR* alterations. Regardless of histology, incidence of these mutations have been found in one third of patients with clinical benefit and in any of patient with non-clinical benefit [28]. In a recent report of five long-term responders mRCC patients treated with mTOR inhibitor, Voss et al. found genomic alterations with activating effect on mTOR signaling in two genes, *TSC1* and *MTOR*, offering plausible explanation for exceptional treatment response [29]. *TSC1* and *TSC2* may be screened as predictive biomarkers of everolimus response in RCC patients who progressed on VEGF-targeted therapy.

Identification of specific mutations or a set of mutations associate with an aggressive tumor behavior such as *SETD2* (15% of ccRCC)*, PBRM1* (40%)*, BAP1* (15%), *KDM5C* (7%), *MTOR* (5–6%), could be relevant to clinicians considering using liquid biopsy to guide treatment of mRCC [30,31,32].

Sporadic papillary type 1 RCC is associated with *MET* gene alteration, whereas sporadic papillary type 2 RCC is characterized by *CDKN2A*, *SETD2*, *NF2*, *CUL3*, *TERT* mutations, gains of chromosomes 7, 12, 16, and 17 [33]. 

Papillary type 2 RCC can occur in patients with germline mutation in the fumarate hydratase (*FH*) gene associated with the hereditary leiomyomatous and RCC syndrome (HLRCC) [34].

Al-Qassab and colleagues interrogate these prognostic genes along with other commonly mutated genes in RCC creating a panel of 14 genes. Twenty out of 30 preoperative RCC patients had detectable GA, comprehending nonsynonymous, frameshift, stopgain, or splice site mutations, in all genes assayed. Even patients with early stage disease and with 1.1 × 0.7 × 0.5 cm^3^ mass had detectable mutations in ctDNA [35].

## 5. Quantitative Analyses of ccfDNA

Circulating cell-free DNA refers to fragments of acellular nucleic acids of various length detectable in almost all body fluids, including blood, released by apoptotic or necrotic cells. Elevated levels of ccfDNA have been detected in patients with acute blunt trauma, burn victimizes, sepsis, myocardial infarction and in cancer patients [36,37,38]. Extraction of ccfDNA from the plasma and its quantification do not consent to identify which DNA fragments derived from cancer cells or from a necrotic inflammatory process. PCR detection methods can detect down to a single targeted molecule present in a sample with limitation of not being able to detect larger structural variant present in ctDNA. For this reason, parallel next generation sequencing method is used to determine the fraction of tumoral DNA in ccfDNA.

Diagnostic value of ccfDNA was showed measuring ccfDNA levels of healthy individual comparing to ccfDNA in patients with RCC [39]. Measuring ccfDNA fragments derived from cell apoptosis and those derived from cell necrosis, Hauser et al. developed a test with sensitivity of 68–57% and specificity of 70.4–81.5% respectively [40].

CpG island hypermethylation of ccfDNA in patients with RCC is a potential diagnostic biomarker. Methylation levels of CpG islands of *RASSF1A*, *FHIT*, and *APC* genes has been detected in ccfDNA of renal cancer patients [41]. Combined analysis of methylation frequency of multiple genes reach a sensitivity of 62.9% and a specificity of 87%. DNA hypermethylation of *APC* gene also correlated with advanced tumor stage [42]. Jung and colleagues have recently tested *SHOX2* mRNA expression in RCC tissues and *SHOX2* gene body methylation quantitatively in circulating cell-free DNA. Results showed *SHOX2* methylation in tissue and plasma strongly correlates with a high stage disease and risk of death after surgery [43].

ccfDNA is also a potential surveillance biomarker for disease recurrence in patient with localized disease. Postoperative recurrence could be monitored quantitatively with ccfDNA instead with a specific genetic test. The pre-surgical level of plasma ccfDNA in patients with metastatic clear cell RCC was significantly increased compared to patients with localized ccRCC or controls.

Patients with a high ccfDNA value had a significantly higher recurrence rate than those with a low plasma ccfDNA level before and after nephrectomy (*p* = 0.018) [44]. Moreover, quantification of plasma ccfDNA permit to predict therapeutic efficacy of sorafenib on mRCC. Quantitative real-time PCR at six different time-points was performed to analyze concentration of ccfDNA in mRCC patients receiving sorafenib. Patients with remission or stable disease had a significant lower level of ccfDNA compared to patients in progression [45].

Yamamoto et al. analyzed ccfDNA and ctDNA of RCC patients and founded that presence of ctDNA and fragmentation of ccfDNA were significantly associated with poor cancer specific survival. Thus, positive ctDNA was associated with a higher proportion of ccfDNA fragments and with shorter fragment sizes of ccfDNA demonstrating that also fragment size may have a clinical utility as biomarkers [46].

## 6. New Techniques

Although liquid biopsy is gaining prominence in the clinical scenario, certain settings as, for instance, cancer screening and the detection of minimal residual disease after treatment could not benefit of this noninvasive technique due to the limits of the current ctDNA detection methods. In particular, the distinction between ccfDNA and ctDNA require a high sensitive technique, free of technical artefacts at sustainable costs. To this purpose, a Canadian group developed a new sensitive, immunoprecipitation-based protocol to analyze the methylome of low quantities of ccfDNA. They found that the recovery of cancer-specific differentially methylated regions (DMRs) could improve sensitivity of ctDNA detection with a low-cost, high-efficiency method [47].

## 7. Comparison with CTC Assay

**Figure 2:**
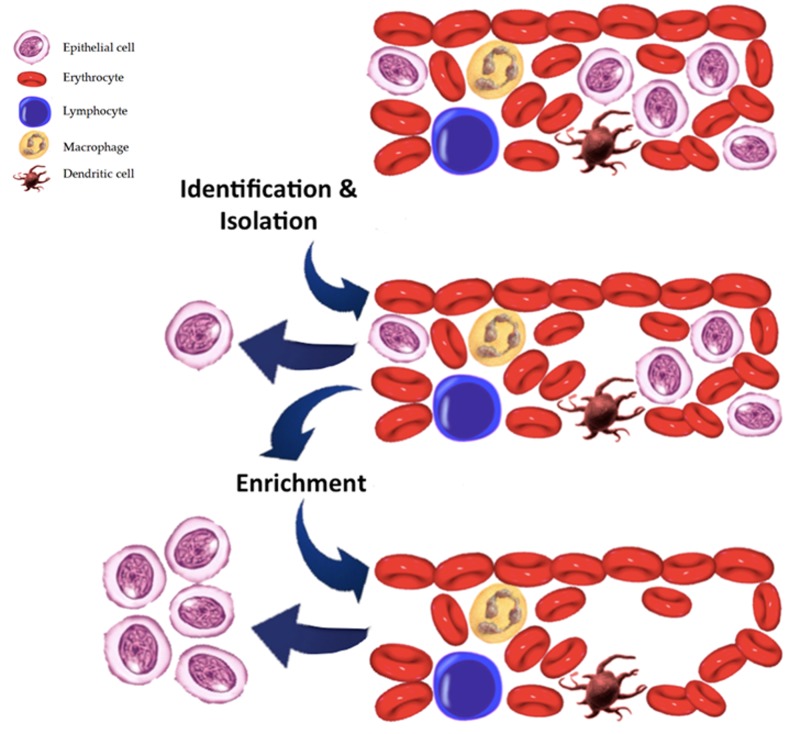
Identification and enumeration of circulating tumour cells.

Nucleic acids have a half-life in the circulation ranging from 15 minutes to several hours, are more stable than cells or RNA and technically easier to isolate than CTCs [48] (Figure 2).

CtDNA represent a pool of cells, give an insight in the repertoire of genetic tumor alterations and on the number and properties of subclones. However, ctDNA in most cases requires *a priori* knowledge of the target of interest and not all DNA mutations are expressed. It has to be isolated from a vast amount of wild type ccfDNA, also detected in healthy individuals in minor levels compared to cancer patients. As response-to-therapy biomarker, it is still unclear if cancer cells shed ctDNA because they are dying from therapy or if because they are resistant to therapy. Cytotoxic chemotherapy induces leukocyte and erythrocyte apoptosis, which leads to a release of ccfDNA into plasma, a potential confounder factor. On the other hand, CTCs are either apoptotic or viable, but only viable CTCs are important for developing metastases and are therefore of special attention.

In metastatic breast cancer, ctDNA is more sensitive than Cell Search CTC Assay, a better biomarker in monitoring tumor dynamics showing a greater correlation with changes in tumor burden, than did CA 15-3 or circulating tumor cells [49]. On the other hand, CTC can be used for functional assays (DNA, RNA, protein) and can be cultured to evaluate drug resistance in vitro or in vivo. Intact CTCs are rare events, isolation is technically challenging.

Current systems of isolation rely on methods based on physical differences between hematopoietic cells and tumor cells and on immune cytokeratin expression such as the epithelial cell adhesion molecule (EpCAM). Level of expression of cytokeratin is a sampling bias; thus, when the tumor cells go through epithelial mesenchymal transition (EMT), as occur in sarcomatoid changes in RCC [50,51], they lost cytokeratin expression leading to a partial or complete switch to a mesenchymal phenotype and so become undetectable at CTCs analysis. To overcome this bias, Ivonne et al. developed a CTC detection technique based on multi-parameter immunofluorescence microscopy (MPIM) that consist of epithelial markers such as CK or EpCAM and cells with mesenchymal and stem cell-like properties [52].

A new combination of cell surface markers comprising CA9 and CD147 as alternative CTC-detective antigens have been developed for RCC patients and demonstrated significantly higher efficiency compared to the conventional EpCAM-based method [53]. However, certain assays cannot be applicable to ctDNA, such as splice variants, information at single-cell level and functional assays. Analytes i.e. proteins, metabolites and RNA can be found only in CTCs [54,55].

## 8. Conclusions

Application of liquid biopsy in the clinical scenario pave the way for a new research field. While identification of predictive marker, prognostication, classification of molecular subtypes can be accessible using both tissue (solid biopsy) and CTCs/ctDNA, tracking of clonal evolution over time, early identification of resistance mechanisms, monitoring treatment response, detection of recurrence and residual disease can be possible only with liquid biopsy in a non-invasive manner.

Many questions and challenges to implementation of liquid biopsies are currently under investigation along with emerging liquid biopsy analytes (i.e., EVs, cfRNAs, branched- chain amino acids (BCAAs), proteins, tumor- educated platelets. A better understanding of the mechanisms involved in release of liquid biopsy constituents and adoption of developing integrative multidimensional profiling approaches will be crucial to solving these challenges.
